# Achieving Engagement With Services in the Asylum Seeker and Migrant Cohort

**DOI:** 10.1192/bjo.2022.443

**Published:** 2022-06-20

**Authors:** Katie Herwig, Oguzhan Yenice

**Affiliations:** Mersey Care NHS Foundation Trust, Liverpool, United Kingdom

## Abstract

**Aims:**

Once refugees and migrants have accessed mental health services, there are a number of potential barriers to establishing a positive therapeutic relationship with clinicians and engaging patients in treatment. WHO recommend that training programmes can help clinicians to understand and assess mental health difficulties according to different cultural explanatory models of psychological symptoms. This audit aims to explore current training of those who work with asylum seekers and migrant patients, within the Merseycare Early Intervention in Psychosis teams.

**Methods:**

A survey of 11 questions was sent out to all assessors, team managers, care co-ordinators, psychologists and doctors within the Early Intervention in Psychosis teams. Questions were asked regarding demographics of participants, their views of the difficulties and barriers of working with asylum seekers, their current level of training to work with this particular cohort of patients and their willingness to attend such training.

**Results:**

33 results were collected and consisted of a broad range of team members. Only 4 out of the 33 participants had any form of training prior to or during their time working with this cohort of patients and 3 of the 4 who had, stated that it was not specific training. Difficulties highlighted included; language barriers, cultural differences, a lack of understanding of the asylum process and a lack of knowledge for local support. All participants said they would be willing to attend training specific to working with the asylum seeker patient population.

**Conclusion:**

This audit demonstrates that we are far from reaching the World Health Organisation recommendations of cultural training for all who work with asylum seeker and migrant patients. It also demonstrates a felt need amongst staff for training to be provided. Recommendations from this audit is to consider mandatory training for all staff, including; cultural training, guidance on use of interpreters and awareness of external support agencies.

